# The Impact of the Skin Microbiome and Oxidative Stress on the Initiation and Development of Cutaneous Chronic Wounds

**DOI:** 10.3390/antiox14060682

**Published:** 2025-06-04

**Authors:** Manuela Martins-Green, Jane Kim, Klara Aziz

**Affiliations:** Department of Molecular, Cell and Systems Biology, University of California Riverside, Riverside, CA 92521, USA

**Keywords:** skin microbiome, diabetes, comorbidities, chronic wounds, oxidative stress, bacteria

## Abstract

Wound healing is a very complex process composed of several phases in which precise events occur, both temporally and specially. However, when these processes go awry, biofilm-forming bacteria become installed in the healing tissue, and the patient has comorbidities, so the wounds do not heal and become chronic. In this review, we describe the importance of high levels of oxidative stress (OS) and bacteria from the skin microbiome in the initiation and development of chronic wounds. The skin microbiome is diverse in humans, and its composition is dependent on the environment in the specific areas of the body. OS is critical for wound healing as it stimulates the immune system to destroy pathogens and secrete cytokines and growth factors that stimulate healing. When OS levels become high in the wound and the bacteria of the skin install themselves in the wound, chronicity ensues. However, neither OS nor the bacteria of the skin alone can initiate chronicity. However, when present together, chronic wounds develop. Given the complexity of chronic wound initiation, developing treatment for these wounds has been difficult. Here, we also discuss the challenges of treating chronic wounds and offer a potential sequence of approaches to treating these wounds after debridement.

## 1. Background in Wound Healing

Cutaneous wounds are wounds of the skin. These wounds occur when the skin is injured mechanically, chemically, or by extreme temperatures. The healing of cutaneous wounds is critical because if healing does not occur in a timely manner, wounds may become infected, and if the infection becomes systemic, the patient could die. During the normal healing of a cutaneous wound, the wound goes through several sequential, overlapping, and timely stages that culminate with the closure of the wound and the replacement of the original damaged tissue with a scar [1,2,3,4,5]. The first of four overlapping phases of healing is hemostasis, in which a clot consisting of fibrin and platelets forms to stop the bleeding, provides a temporary matrix for cell migration, and protects the exposed tissue. The platelets release factors that chemoattract leukocytes to the injured tissue and in this manner stimulate the inflammatory phase. Neutrophils are the first type of leukocytes to appear in the wound and are primarily attracted by the chemokine IL-8. These cells produce reactive oxygen species (ROS) and enzymes that kill pathogens and in this manner deter the infection of the wound tissue. Neutrophils also contain cationic proteins, hydrolases, and an acidic pH in their phagocytic vacuoles which kill bacteria in hypoxic and anoxic environments. Moreover, neutrophils produce matrix metalloproteinases to digest the components of dead cells and the damaged extracellular matrix (ECM). Following neutrophils infiltration, monocytes, which are attracted by a variety of factors including the chemokine monocyte chemoattractant protein, arrive at the wound site and differentiate into pro-inflammatory macrophages. These cells remove dead neutrophils and clean the wound bed of cellular debris caused by neutrophils as they kill and phagocytose pathogens. These pro-inflammatory macrophages are followed by anti-inflammatory macrophages that secrete growth factors and cytokines that promote healing. During the proliferative phase, fibroblasts in the wound tissue secrete ECM molecules that provide structural and biochemical properties to the newly formed granulation tissue, which is named so because of its microscopic granular appearance. During this phase, keratinocytes proliferate and migrate over the granulation tissue to close the wound. Simultaneously, vascular endothelial cells in the granulation tissue form microvessels that bring nutrients and oxygen to support the development of the healing tissue. Moreover, lymph endothelial cells form lymphatic microvessels which are critical for the drainage of fluid produced during healing. During this phase, macrophages continue to secret enzymes that remodel the ECM and produce cytokines and growth factors that induce fibroblast differentiation into myofibroblasts. During remodeling, the last phase of wound healing, the extra cells undergo apoptosis, and the excess ECM produced during the development of the healing tissue is removed by phagocytes to remodel the wound tissue and generate a scar. This scar can become excessive in certain kinds of abnormally healing wounds and cause significant discomfort, but they do heal and do not develop a biofilm [4].

In addition to the normal processes involved in the host component of acute cutaneous wound healing, it is important to consider that the other component important for healing is the microbiota of the skin. Indeed, it has been shown that a bacterial load of up to 10^5^ colony-forming units (CFUs)/g of viable tissue is needed for proper healing [2,6,7,8]. These pathogens form polymicrobial communities that release molecules that attract neutrophils to the wound and initiate the processes of healing.

It is well known that alterations in any of these complex and highly controlled processes of normal healing either temporally or spatially prevent wounds from healing properly. They may develop excess healing (e.g., [4]) or not heal at all, in which case they become chronic (e.g., [5]). A wound is considered chronic when it is still not healed after one month of standard treatment has started [9].

It has been reported that in the US alone, ~8.5 million people are impacted by chronic wounds and cost ~USD 28–90 billion/year. Moreover, these costs do not account for the pain and suffering that patients endure, both psychologically and physically [10,11]. Chronic wounds become infected, and when that infection becomes chronic, they can develop microbial biofilms, a situation that becomes very serious not only because biofilms are very difficult to treat but also because systemic infections can arise and lead to death. Furthermore, uncontrolled infections in wounds of the extremities can lead to amputation [12,13].

In addition, chronic wounds develop primarily in people who have comorbidities such as diabetes, cardiovascular disease, poor nutrition, and obesity. According to a recent report, gender is a critical parameter to take into consideration when evaluating patients for treatment [14]. Metabolic disorders are more common in males with ulcers and autoimmune diseases in females with ulcers. Therefore, it is critical that the comorbidities in males and females be identified so that appropriate treatments for these comorbidities are effectively used. Moreover, age is also important for the development of chronicity. People are more likely to develop chronic ulcers after 60 years of age, and the older the patient is, the less likely the ulcers are to heal [15].

Many treatment protocols have been developed during the past decades. Wounds are debrided; infection and pain are controlled; standard treatments are performed, and dressings are applied. Some of the treatments used currently involve the application of various types of grafts; many products contain cells which are designed to resemble normal skin and hyperbaric oxygen treatment, but so far, none have been truly effective. Therefore, more research is needed to understand how chronicity initiates so that new approaches to treatment can be developed to switch the path of chronic wounds to the path of healing wounds. This is particularly important when wounds are debrided to remove damaged tissue and biofilms, and a new margin of “healthy” skin is now present; an optimal outcome would be that the wound can now be treated to heal as opposed to remaining chronic. However, the biofilm often returns within 72 h and does so as aggressively as before [16].

To increase the probability for a chronic wound to heal, it is important to create a microenvironment that will favor the processes of acute wound healing. Furthermore, it is critical that the wound microenvironment is unfavorable for chronic infection and biofilm development. Indeed, to be able to change a wound that is becoming chronic into a wound that can heal, we need to understand fully the pathophysiology of the initiation of wound chronicity. Because these types of experimentation cannot be conducted in humans with chronic wounds, it is imperative that animal models be used to study how wounds acquire and develop chronicity.

## 2. Diversity of Skin Microbiota in Humans

The human body is not only composed of cells, tissue, and organs that govern its physiology, but humans also live in a constant relationship with microorganisms that, for the most part, establish a balanced interaction with mutual benefits. However, when this balance is disrupted, disease can ensue. Microorganisms are primarily abundant in the gut, hair follicles, glands, mucosa, and the skin.

In an average size human adult, the skin covers ~2 m^2^ of the surface, making it the largest tissue in the body. Skin has evolved into a barrier that protects the internal organs from physical, chemical, and biological attacks from the environment and pathogens. When a skin injury occurs, the body relies on the complex process of cutaneous wound healing, as described above, to properly close the damaged area and restore the protective antimicrobial and barrier functions of the skin. The microbiota of the skin is complex and diverse (Table 1: taken from *Microorganisms* 2025, 13, 868. 10.3390/microorganisms13040868) [17,18,19]. Trillions of microbes across the bacterial and fungal phyla use aerobic and anaerobic metabolic pathways to survive. In addition, viruses are also present in the skin. The collective genome of the microbiota constitutes the microbiome and contains wide genomic and proteomic profiles that are specific for a specific location of the body [17,18,20].

One of the approaches scientists have taken to identify wound microbes was by isolating the pathogens from the wound and culturing them under artificial laboratory conditions. However, these conditions have limitations because they might not be appropriate for a particular microorganism to grow. This is particularly true for anerobic populations, limiting the ability to identify and describe the whole microbiota [17,21,22]. In the last decade, advances in high-throughput DNA sequencing help identify many microbes in normal healthy human skin and have also characterized the microbiome of wounded skin [23,24,25,26].

Using this high-throughput DNA sequencing method, it has been shown that the bacteriome of human skin is composed of major bacterial families such as *Actinobacteria*, *Firmicutes*, *Proteobacteria*, and *Bacteroidetes*, with Actinobacteria being more abundant on the skin [18]. Other contributors at the genera level to the skin microbiome are *Corynebacterium*, *Propionibacterium*, and *Staphylococcus* [18]. However, different bacteria populate different areas of the skin because the skin has different characteristics depending on the location and composition of the appendages. Areas with abundant sebaceous glands are predominantly populated by the *Propionibacterium acnes* and *Staphylococcus* species (for example, in the face). *Propionibacterium acnes* is a common skin commensal bacterium that produces multiple lipases that degrade lipids in the skin [17]. In areas that are moist, such as the axilla, the predominate members of the microbiota are the *Propionibacterium*, *Corynebacterium*, and *Staphylococcus* species. On the palm of the hand, *Propionibacterium*, *Streptococcus*, *Staphylococcus*, *Corynebacterium*, and *Lactobacillus* are detected [27,28].

However, when in drier areas of the skin or when sebum is not produced, the bacterial populations are mixed [17,27]. On the forearm, *Propionibacterium*, *Corynebacterium*, *Staphylococcus*, *Streptococcus*, *Acinetobacter*, and *Finegoldia* are present [29]. On the skin surrounding the ankles, bacteria such as *Pseudomonas*, *Corynebacterium*, *Staphylococcus*, *Chryseobacterium*, *Acinetobacter*, *Methylophilus*, *Acidithiobacillus*, *Segetibacter*, *Wautersiella*, and *Psychrobacter* can be found [17]. On the feet, the microbiota contains primarily bacteria from the genera *Staphylococcus*, *Acinetobacter*, *Kocuria*, *Corynebacterium*, and *Micrococcus* [17]. Using 18S rRNA as a marker, fungi species were also found in the skin [30]. Fungi are lipophilic microbes that live in areas of the skin that produce significant levels of sebum. Finally, the role of viruses and their function as microbes in the skin are still under investigation.

## 3. Bacterial Microbiome in Human Chronic Wounds

Considerable attention is being given to study the role of the microbiota in normal healing and skin-related infections, disorders, and diseases [31,32,33]. Host–microbe interactions are particularly relevant in wound healing because the microbiome play an important role in the healing or non-healing of wounds. It is known that microbial presence in the wound is critical for proper healing. This is because microbes stimulate neutrophil and macrophage infiltration, which clears the wound of pathogens and activate normal healing.

Understanding how different skin pathogens colonize specific areas of the body could provide insights into the pathophysiology of cutaneous infections and may explain how we can modulate the microbiota and prevent wound chronicity [34]. High oxidative stress (OS) in chronic wounds provides the environment that can support the survival of pathogens that become virulent and form a biofilm. Multiple studies in humans cutaneous chronic wounds show that these wounds are colonized by many bacterial species [35,36,37,38,39,40,41,42,43,44,45]. By performing genomic analysis of several types of human chronic wounds, it has been shown that no single microorganism has been identified to be the causal agent of infections/biofilms in chronic wounds, adding to their complexity. Below, we describe the bacterial composition for the three major types of chronic wounds, namely diabetic foot ulcers, venous ulcers, and pressure ulcers.

*Diabetic foot ulcers*: Foot ulcers are primarily found in diabetic people hence the name of diabetic foot ulcers (DFUs); they are high-risk wounds that result in a high incidence of hospitalization, and patients with these wounds are at an increased risk of amputation [12,13]. Because of diabetic neuropathy in the feet, these patients have very poor sensation and cannot feel the pressure, pain, and discomfort caused by the injury and do not discover the wound until it develops. These wounds have poor peripheral blood circulation, which results in impaired oxygenation of the wound area [46]. Impaired wound circulation also increases cell death and eschar tissue and decreases fluid drainage, suggesting damaged lymphatic vessels, and these wounds frequently present with bad odor. Without proper treatment, they form ulcers [46]. DFUs are colonized by a variety of bacteria species which include *Acinetobacter*, *Bacillus*, *Enterobacter*, *Escherichia*, *Cutibacterium*, *Enterococcus*, *Pseudomonas*, and *Staphylococcus* [44,46,47,48,49,50,51,52,53]. These bacteria, alone or in combination, can form a biofilm, making these wounds very difficult to treat and heal.

*Venous ulcers:* Venous ulcers form because of chronic venous insufficiency; the calf muscle fails to pump blood, while retrograde blood flux does not occur because the valves in the calf are unable to maintain proper blood pressure [43,54,55,56,57,58,59]. These wounds, in general, have an irregular margin, and the wound bed of the ulcer has a fibrous layer mixed with the granulation tissue [56]. Fibrinogen in the tissue, in the presence of thrombin released by leaky blood vessels, polymerizes into fibrin, which deposits in the form of pericapillary fibrin ‘cuffs’ [56,57]. These ‘cuffs’ are thought to prevent the proper movement of leukocytes to the wound site and, more importantly, prevent the proper diffusion of oxygen and nutrients into the tissue, resulting in increased hypoxia. Venous ulcers develop 95% of the time on the lower portion of the leg from the calf to the ankle and are more prevalent in males and/or people with a history of vessel obstruction, inactivity, a high body mass index, and a previous history of ulcers in the family [43,53,57,59]. Venous leg ulcers are predominantly infected with *Staphylococcus aureus*, *Corynebacterium*, *Pseudomonas aeruginosa*, *Serratia*, and *Streptococci* bacteria [34,53].

*Pressure ulcers:* Pressure ulcers occur in the skin or underlying tissues because of direct perpendicular pressure alone or in combination with shear forces over bone protrusions [43,60]. With age, the tissue underlying the skin, as well as the skin itself, loses elasticity, causing decreased resistance to pressure and resulting in tissue maceration and cell death. Moreover, the lack of elasticity can give rise to blisters that can burst and cause an open wound. Age is an important risk factor for pressure ulcers, especially in elderly and disabled patients, who are in a wheelchair or are confined to a bed. Other risk factors include cardiovascular diseases, malnutrition, paralysis, and incontinence. In addition, people with diabetes mellitus have an increased risk of developing pressure ulcers. Pressure ulcers are often colonized by many bacteria species [60,61,62]. Common bacteria include *Streptococcus*, *Peptoniphilus*, *Anaeroccocus*, *Gemella*, *Corynebacterium*, Enterobacteraceae, *Anaerococcus*, *Acinetobacter*, *Finegoldia*, *Micrococcs*, *Enhydrobacter*, *Prevotella*, *Lachnospiraceae*, *Blautia*, *Lactobacillus*, *Staphylococcus*, and *Enterococcus*, with the latter two found to be the most abundant in patients with these ulcers [61].

## 4. Oxidative Stress, Microbiome of the Skin, and Biofilm Formation

**Figure 1:**
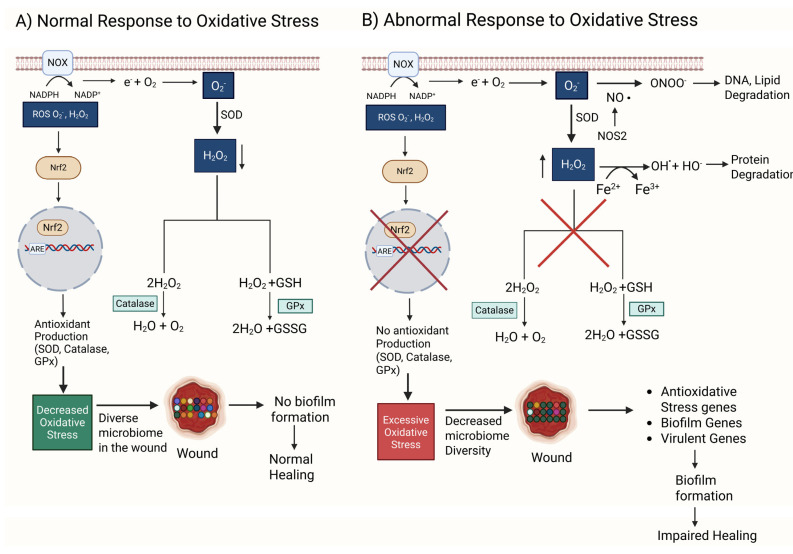
**A comparison between normal and abnormal responses to oxidative stress (OS).** (**A**) NOX oxidizes nicotinamide adenine dinucleotide phosphate (NADPH) to NADP^+^, generating an electron that reduces O_2_ to form a reactive superoxide anion O_2_^−^. O_2_^−^ is used by SOD to produce H_2_O_2_, a less reactive oxidative stress (ROS) molecule. H_2_O_2_ is converted to water and oxygen by catalase, an antioxidant enzyme. H_2_O_2_ and glutathione (GSH) are converted to water and glutathione disulfide (GSSG) by glutathione peroxidase (GPx), another antioxidant enzyme. Low levels of ROS can act as signaling molecules that activate Nrf2, a transcription factor that binds to DNA sequences such as ARE (antioxidant response element) to enhance the transcription of antioxidant genes. Under these conditions, the wound microbiome stays diverse. A diverse microbiome in the wound prevents biofilm formation and promotes normal wound healing. (**B**) An abnormal response consists of malfunctioning GPx and catalase, increasing the amount of O_2_^−^ and H_2_O_2_ and resulting in redox imbalance and excessive oxidative stress. Under these conditions, O_2_^−^ when in the presence of nitric oxide (NO) and NOS produces ONOO^−^, an anion that damages DNA and lipids. Simultaneously, increased levels of H_2_O_2_ in the presence of ferrous ions produce HO^•^ radicals through the Fenton reaction. HO^•^ damages proteins. Together, HO^•^ and ONOO^−^ result in DNA, protein, and lipid degradation, which leads to cell dysfunction and eventually cell death. Excessive OS also causes the wound microbiome to be controlled by biofilm-producing bacteria, reducing the diversity of the microbiome. Unlike the other bacteria, biofilm bacteria suppress the effects of OS by turning on antioxidative stress genes, biofilm-forming genes, and virulent genes. The biofilm in the wound causes impaired healing. Red X indicates eliminated pathway.

OS is present in cells and tissues when there is an imbalance between the levels of antioxidant molecules and/or antioxidant enzymes that neutralize and remove reactive oxygen species (ROS). ROS are highly reactive forms of O_2_-derived radicals or atoms/molecules that contain one or more unpaired electrons. A superoxide anion (O_2_^•−^) is one of the most damaging ROS. Inside the cell, nicotinamide adenine dinucleotide phosphate (NADPH) oxidase subunits assemble in the membrane, and in the presence of injury/stress, this enzyme transfers electrons from NADPH to O_2_, giving rise to a superoxide anion O_2_^•−^ (Figure 1). In addition, O_2_^•−^ is also produced during the mitochondrial electron transport chain because small amounts of O_2_ leak from complexes I and III during the oxidative phosphorylation chain reactions. O_2_^•−^ is also produced by phagocytic cells, such as neutrophils, in the process of killing bacteria. These leukocytes are very effective in killing bacteria via the respiratory/oxidative burst, a defense mechanism that involves the production of high levels of O_2_^•−^ and H_2_O_2_ via NADPH oxidase into bacteria-containing phagosomes, leading to the killing of these infectious agents [63].

O_2_^•−^ is dismutated to H_2_O_2_ spontaneously or by superoxide dismutase (SOD) and then broken down into H_2_O by glutathione peroxidase (GPx) and into H_2_O and O_2_ by catalase, two very effective antioxidant enzymes present in cells and tissues. Moreover, NRF2 is a transcription factor turned on by ROS which then activate the expression of antioxidant enzymes (Figure 1A). GPx and catalase decrease ROS and provide an environment in which the microbiome in the wound remains diverse, resulting in a biofilm not forming and healing occurring normally.

H_2_O_2_ is a major signaling ROS during wound healing; it is easily synthesized and degraded; it moves readily through cell membranes and tissues because it is uncharged. Moreover, it can have a long half-life and is selective in the molecules it reacts with. At low concentrations (10 µM), it attracts leukocytes to the wound site and stimulates these immune cells to initiate the processes of healing. At moderate concentrations (100 µM), it stimulates the expression of basic fibroblast growth factor (bFGF), a growth factor that stimulates fibroblasts to proliferate and migrate, and increases the expression vascular endothelial growth factor (VEGF), which stimulates angiogenesis. H_2_O_2_ also stimulates signaling by transforming growth factor beta 1 (TGFβ1), which leads to increased chemotaxis of keratinocytes and the stimulation of ECM production [64,65].

If this system of factors/antioxidants that work during normal healing does not function correctly or comorbidities such as hyperglycemia are present, excessive OS builds in the tissue, causing significant damage (Figure 1B). When in the presence of ferrous ions from leaky vessels, H_2_O_2_ can produce both hydroxyl anions (OH^−^) and radicals (^•^OH), whereas O_2_^•−^ when is in the presence of nitric oxide (NO) generated by nitric oxide synthetase (NOS) can generate the anion ONOO^−^. These anions and radicals all damage DNA, proteins, and lipids, leading to cell dysfunction and death (Figure 1B). Simultaneously, Nrf2 is inhibited, and therefore, antioxidant enzymes are not produced, leading to an increase in OS. A high level of OS not only causes cell and tissue damage but also allows biofilm-forming bacteria to survive, while the “good” bacteria succumb to high levels of oxidative stress, leading to biofilm formation and wound chronicity (Figure 1B) [66,67].

**Figure 2:**
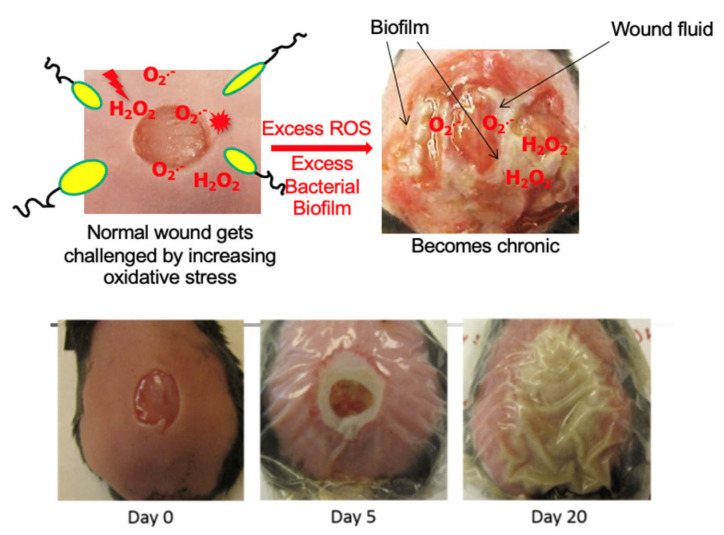
**Chronic wound model in *db*/*db* mice [68].** A wound is created at day zero and immediately treated with antioxidant enzyme inhibitors. This results in a wound that becomes fully chronic by 20 days after wounding. These wounds produce exudate that contains bacterial biofilms.

We have previously shown in our diabetic model of chronic wounds [68,69] that the microbiome of the skin is critical for the development of a biofilm in the wound and that the biofilm is necessary albeit not sufficient for chronic wound development [66]. Using the *db/db* mouse model for chronic wounds that we developed (Figure 2) where pathogenic biofilms develop naturally, we sequenced the bacterial rRNA internal transcribed spacer (ITS) gene of the wound microbiome from wound initiation to when wounds are fully chronic. Using this approach, we found that healing wounds were colonized by a highly diverse and dynamic bacterial microbiome that never developed biofilms even though biofilm-forming bacteria were present [66]. Bacteria such as *Cutibacterium acnes*, *Achromobacter* sp., *Delftia* sp., and *Escherichia coli*, which are clinically relevant, were highly associated with healing wounds. These bacteria could serve as indicators of healing, may participate in the processes of wound healing, and prevent pathogenic bacteria from colonizing the wound. In contrast, chronic wounds, which had high levels of OS, had low bacterial diversity and were colonized by several clinically relevant, biofilm-forming bacteria such as *Pseudomonas aeruginosa*, *Enterobacter cloacae*, *Corynebacterium frankenforstense*, and *Acinetobacter* sp. [66].

Probiotic bacteria do not survive high levels of OS, whereas the biofilm-forming bacteria that are otherwise suppressed by the probiotic bacteria in the skin will readily survive by turning on genes that combat OS and other genes that help them make biofilms and become virulent [66,67,70]. We have shown that *Pseudomonas aeruginosa*, in the presence of high levels of OS in the wound, will turn on antioxidative stress genes, biofilm-forming genes, and virulent genes that help it survive in the high OS environment; form biofilms, and contribute in this manner to wound chronicity [70].

## 5. Importance of Treating Oxidative Stress and Biofilm to Heal Chronic Wounds

As described above, wound healing is a very complex process that involves not only the host response to the injury but also the complex skin microbiome. The progression of wound colonization by the skin microbiome from normal healing to infection and chronicity depends not only on the bacteria counts, the species present, and their virulence but also on the levels of OS in the wound tissue. Approximately ~80% of chronic wounds contain biofilms, whereas only ~6% of acute wounds contain biofilms. As shown previously, high levels of OS alone are necessary but not sufficient for chronicity [67]. The same is true for the microbiome [67]. High levels of OS in both the wound and the microbiome are critical for biofilm formation and chronic wound development.

Bacterial biofilms are defined as bacterial cells embedded within an exopolymeric matrix composed of molecules produced by the bacteria as well as DNA, RNA, proteins, and polysaccharides from the dead host cells. When within this matrix, the bacteria become dormant (low metabolism), are protected from host defenses, and are also tolerant to antibiotic treatments. When treating chronic wounds that contain biofilms, it is imperative that treatments be first directed to eliminate OS and the biofilm, followed by treatments that stimulate the healing processes such as the control of inflammation, the stimulation of proliferation, the migration of keratinocytes to close the wound, the migration of vascular endothelial cells to form new microvessels that bring oxygen and nutrients to the wound and lymph endothelial cells to form the lymphatic vessels for the drainage of the wound, and the deposition of ECM molecules by fibroblasts to knit the granulation tissue together.

The most effective way to remove OS is by treatment with antioxidant molecules, and to remove biofilms, debridement is the most effective method. There are many ways that debridement can be performed. Sharp surgical debridement is one the most effective method because it removes both the biofilm and the dead tissue in the wound [9]. However, not all bacteria are removed during debridement; some bacteria that are buried deep in the wound tissue remain and will return as a biofilm within 72 h after debridement [16]. Therefore, debridement must be followed by other treatments to ensure that the bacteria do not return.

Our studies of the bacterial microbiota over time in a *db/db* mouse model of chronic wounds [66,67] suggest that a critical point of treatment to prevent the return of the biofilm is shortly after debridement [66,67,68]. Indeed, many treatments have been used after the debridement of wounds including nanocrystalline silver, sustained release iodine, and topical application of antibiotics/antimicrobials [71,72,73]. Although these small molecules should be successful treatment options because they have low cytotoxicity, can be applied topically, and are inexpensive off the shelf, these treatments are frequently not effective, and the bacteria and biofilm return. Other types of treatments that involve the use of natural products such as curcumin [74,75] and honey [76,77] have also been used but not very effectively. Therefore, new treatments are needed, in particular those involving small molecules that are not expensive and are durable off the shelf.

Two molecules that have shown promise in treating chronic wounds with biofilms are *N*-acetyl cysteine (NAC) and chlorate [68,78]. Using our *db/db* mouse model of chronic wounds [68,69], we have shown that treatment with NAC leads to biofilm dismantling and bacterial cell death and reduces the OS damage to host cells, thus allowing healing to proceed [68]. Mechanistically, we have shown that at a pH below the pKa, NAC causes bacterial cell death by halting protein synthesis [79]. Once the bacteria die and the biofilm dismantles, the wound goes on to heal [68].

Sodium chlorate treatment has been tested in the same mouse model of chronic wounds. Chronic wounds that are only infected by *Pseudomonas aeruginosa* and contain biofilms formed exclusively by this bacterium showed that the treatment of the wounds with sodium chlorate resulted in wounds healing faster than untreated chronic wounds [78]. Because the wound microenvironment is largely devoid of oxygen, *Pseudomonas aeruginosa*, a facultative anaerobe, relies in part on anaerobic metabolism, such as nitrate respiration, to survive in the deep tissue in chronic wounds. While nitrate reductase (nar) typically reduces nitrate to nitrite for *Pseudomonas aeruginosa* metabolism, it can also reduce chlorate to chlorite, the latter of which is a toxic oxidizing agent that kills *Pseudomonas aeruginosa*. Therefore, chlorate can act as a “drug” to specifically eradicate hypoxic/anoxic, nitrate-respiring bacterial populations, which are often tolerant to conventional antibiotic treatments. Once the biofilm begins to dismantle, the wounds progress to healing. In these treated wounds, granulation tissue formation, reepithelization, blood vessel development, and matrix deposition were similar to those of the healing non-chronic wound [78]. These results indicate that there is a possibility of discovering affordable, small molecules with a long shelf life that can be used in treating chronic wounds with biofilms.

## 6. Challenges in Treating Human Chronic Wounds

To date, it is not possible to understand how chronic wounds develop in humans. When presented to the physician, these wounds are already fully chronic, making it impossible to determine how chronicity initiated and how the biofilm developed. This creates an enormous challenge for developing treatments and discovering cures for human chronic wounds.

One of the important considerations when examining a patient is that people with chronic wounds have underlying comorbidities even though not all patients have the same pathophysiology. Many humans with chronic wounds are obese, have diabetes, have poor vascular and lymphatic circulation, have cardiovascular disease, and have poor nutrition, and also, these wounds depend on gender and age. Therefore, treating comorbidities is one the most critical aspects of treating chronic wounds. It is important to put the patient in a physiological condition that allows the tissues to heal.

Different wounds have different etiologies; therefore, they must be treated differently. A pressure ulcer will require a different approach to treatment than a DFU. The sites in the skin where these wounds develop are different; therefore, different microbiomes need to be taken into consideration. One of the problems in determining what is the best treatment for a particular chronic wound is the poor understanding of the cell and molecular processes that go awry during the establishment of chronicity. Therefore, a better understanding of these processes and mechanisms during chronic wound initiation and development and a better understanding of host–microbe interactions could help unravel the pathophysiology of chronic wounds and help decide on treatments.

Recent efforts to understand the pathophysiology of chronic wounds has involved “Omic” studies such as metabolomics, transcriptomics, lipidomics, and proteomics, which are all approaches that have shed some light in the cell and molecular mechanisms that contribute to chronic wounds at different stages. However, because of the nature of the studies in humans, considerable gaps are still present in generating a complete picture of how chronic wounds initiate and develop. Therefore, current therapies based on these findings are often not effective [80]. The situation is complicated by the paucity of preclinical models for studies of chronic wound development, hindering the ability to find treatments that are translatable to humans in the clinic [68,69,80,81,82].

**Figure 3:**
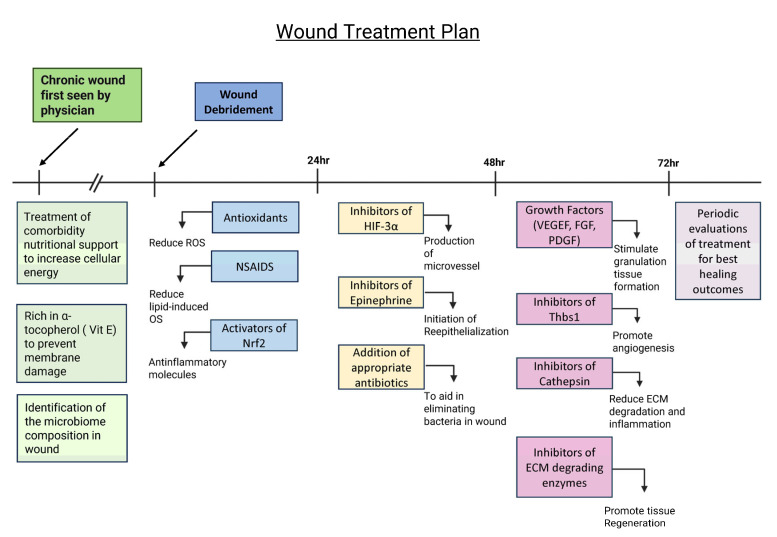
**Potential treatment plan for chronic wounds to promote wound healing.** Treating comorbidities, providing nutritional support, and identifying the microbiome composition prior to debridement are critical to create an environment for proper wound healing. A diet rich in vitamin E and products that produce energy is also necessary for cell function and protection against lipid peroxidation. After wound debridement, the wound should be treated with antioxidants, NSAIDs, and activators of Nrf2 to balance out levels of OS and reduce inflammation. During the first 24–48 h, the wound should be treated with inhibitors of HIF-3⍺ and epinephrine and appropriate antibiotics in order to start angiogenesis and re-epithelialization and reduce the bacterial load. During the 48–72 h period, the wound should be treated with growth factors; inhibitors of thrombospondin (Thbs1), a protein that inhibits angiogenesis and is abundant in chronic wounds; cathepsin; and ECM-degrading enzymes to inhibit matrix degradation. After 72 h, the wound needs to be observed frequently to evaluate healing and determine further course of treatment.

Based on current published findings, we have recently proposed a potential approach to treating chronic wounds after debridement (Figure 3). We propose that it is important to treat the physiological problems related to the comorbidities these patients have prior to and during wound treatment. Patients should also be put on a diet that is rich in products that stimulate an increase in the levels of adenosine triphosphate (ATP) to ensure that the cells have sufficient energy to function and is rich in α-tocopherol (Vit E) to decrease lipid peroxidation and cell membrane damage, and patients should also take antioxidants to reduce OS. In addition, it is very important to identify the composition of the wound microbiome and determine whether a biofilm is present so that appropriate biofilm-dismantling molecules and antibiotic/antimicrobial agents can be applied.

Once the comorbidities are being treated and the microbial composition of the biofilm is known, during the first 24 h after debridement, the wound may be treated with the following (1) antioxidant molecules, such as NAC, that eliminate local ROS with non-steroidal anti-inflammatory drugs (NSAIDs) to decrease OS caused by pro-inflammatory lipid molecules and an activator(s) of nuclear factor erythroid 2-related factor 2 (Nrf2), a transcription factor that stimulates genes that express anti-inflammatory molecules [83,84]. During the first 48 h, the wounds should be treated with an inhibitor(s) of hypoxia-inducible factor (HIF)-3α, a transcription factor that counteracts HIF-1α and HIF-2α transcription activity but does not induce specific transcription activity; therefore, it has a negatively effect on the gene expression of factors turned on by HIF -1α and HIF-2α such as vascular endothelial growth factor (VEGF), platelet-derived growth factor (PDGF), and FGF, which are needed for angiogenesis and granulation tissue formation [85]. In addition, the wounds should be treated with inhibitors of catecholamines in particular inhibitors of epinephrine to allow for re-epithelialization and with appropriate antibiotics dictated by the microbiome composition [86].

At 48–72 h after debridement, the wound should be evaluated for a second phase of treatment. If the biofilm has not returned, consideration should be given to the use of growth factors that stimulate granulation tissue formation such as PDGF and VEGF, as well as inhibitor(s) of thrombospondin (Thbs1), which is often elevated in chronic wounds, to allow angiogenesis to occur. In addition, inhibitors of cathepsins and other ECM-degrading enzymes should also be applied to decrease matrix degradation. After 72 h, this treatment should be evaluated periodically to determine the best way to continue to promote granulation tissue formation and re-epithelialization to close the wound. This approach is novel, and therefore, the treatment approach proposed here is at the forefront of the field of study.

## 7. Summary and Conclusions

Normal wound healing involves a series of complex processes that are highly regulated (temporally and spatially) and influenced by endogenous and exogenous factors. Chronic wounds are associated with comorbidities and develop when one or more of the healing processes are altered and when pathogens establish themselves in the wound tissue and create a chronic infection and/or form biofilms. Because the composition of the microbiome of the skin varies with the area of the body, it is important to take that into consideration when treating these wounds. Oxidative stress and the microbiome are both necessary and sufficient to initiate wound chronicity, and as chronic wounds develop and a biofilm forms, the microbiome becomes less diverse. Moreover, chronic inflammation, a lack of microvessels, and poor granulation tissue formation occur in chronic wounds, and re-epithelialization to close the wound does not occur. Therefore, because of the complexity involved in the initiation and development of these wounds, in order to treat them, it is necessary to ensure that the patients are healthy and that the microenvironment in the wound is conducive to healing while being unfavorable to the growth and establishment of pathogens. To better achieve this goal, we need to continue to understand the pathophysiology of human chronic wounds, how the microbiota influences the healing processes, and how interactions between the host and pathogens result in chronicity.

## Figures and Tables

**Table 1:** Diversity of the skin microbiome according with skin properties.

Skin Sites and Physiology	Alpha Diversity	Beta Diversity	Microbial Composition
**Dry** (hypothenar palm, volar forearm)	High	High interpersonal variation	*Actinobacteria* *(Propionibacterium 13% and* *Corynebacterium 15%)* *Firmicutes, Proteobacteria (41%) and* *Bacteroidetes (14%)*
**Moist** (Nare, antecubital fossa, inguinal crease, popliteal fossa)	Low	Low	*Colonized predominantly by Firmicutes like* *Staphylococcus (21%), Corynebacterium spp.* *(28%), and Proteobacteria (26%)*
**Sebaceous** (check, glabella, external auditory canal, occiput, back)	Lower	lower	*Colonized predominantly by Propionibacterium spp. (46%) and Staphylococcus (16%)*
